# Glycome profiling by lectin microarray reveals dynamic glycan alterations during epidermal stem cell aging

**DOI:** 10.1111/acel.13190

**Published:** 2020-07-18

**Authors:** Lalhaba Oinam, Gopakumar Changarathil, Erna Raja, Yen Xuan Ngo, Hiroaki Tateno, Aiko Sada, Hiromi Yanagisawa

**Affiliations:** ^1^ Life Science Center for Survival Dynamics Tsukuba Advanced Research Alliance (TARA) University of Tsukuba Tsukuba Japan; ^2^ Ph.D. Program in Human Biology School of Integrative and Global Majors University of Tsukuba Tsukuba Japan; ^3^ Graduate School of Comprehensive Human Sciences University of Tsukuba Tsukuba Japan; ^4^ International Research Center for Medical Sciences (IRCMS) Kumamoto University Kumamoto Japan; ^5^ Cellular and Molecular Biotechnology Research Institute National Institute of Advanced Industrial Science and Technology Tsukuba Japan; ^6^ Faculty of Medicine University of Tsukuba Tsukuba Japan

**Keywords:** epidermal stem cells, glycosylation, lectin microarray, mannose, sialylation, skin aging, stem cell aging

## Abstract

Aging in the epidermis is marked by a gradual decline in barrier function, impaired wound healing, hair loss, and an increased risk of cancer. This could be due to age‐related changes in the properties of epidermal stem cells and defective interactions with their microenvironment. Currently, no biochemical tools are available to detect and evaluate the aging of epidermal stem cells. The cellular glycosylation is involved in cell–cell communications and cell–matrix adhesions in various physiological and pathological conditions. Here, we explored the changes of glycans in epidermal stem cells as a potential biomarker of aging. Using lectin microarray, we performed a comprehensive glycan profiling of freshly isolated epidermal stem cells from young and old mouse skin. Epidermal stem cells exhibited a significant difference in glycan profiles between young and old mice. In particular, the binding of a mannose‐binder rHeltuba was decreased in old epidermal stem cells, whereas that of an α2‐3Sia‐binder rGal8N increased. These glycan changes were accompanied by upregulation of sialyltransferase, *St3gal2* and *St6gal1* and mannosidase *Man1a* genes in old epidermal stem cells. The modification of cell surface glycans by overexpressing these glycogenes leads to a defect in the regenerative ability of epidermal stem cells in culture. Hence, our study suggests the age‐related global alterations in cellular glycosylation patterns and its potential contribution to the stem cell function. These glycan modifications detected by lectins may serve as molecular markers for aging, and further functional studies will lead us to a better understanding of the process of skin aging.

## Graphical abstract

Using lectin microarray, a platform for high‐throughput glycome analysis, we demonstrate that epidermal stem cells undergo an age‐related glycome shift from high mannose‐ to sialylated complex‐type N‐glycans. These glycan alterations are detected by lectin probes, rHeltuba and rGal8N, which serve as potential biomarkers of skin aging.

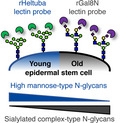

## 1 INTRODUCTION

The epidermis is the first barrier of our body that protects us from infection and dehydration. The epidermis consists of the interfollicular epidermis (IFE) and its appendages (hair follicles: HFs, sebaceous and sweat glands) and is replenished by distinct populations of stem cells (Gonzales & Fuchs, 2017; Rognoni & Watt, 2018). The IFE is renewed by stem cells located in the basal layer, which give rise to stratified squamous epithelium. HF stem cells reside in a specialized structure, called the bulge, and contribute to cyclic regeneration of HFs. Stem cells in the IFE and HFs are largely independent of each other during homeostasis, but they possess plasticity to change their fates in response to injury (Gonzales & Fuchs, 2017; Rognoni & Watt, 2018). The epidermis is separated from the dermis by the basement membrane enriched in the extracellular matrix, which regulates stem cell property and fates (Chermnykh, Kalabusheva, & Vorotelyak, 2018; Watt & Fujiwara, 2011).

An age‐related decline in tissue regeneration and function could be attributed to an impaired stem cell function, a theory known as “stem cell aging” (López‐Otín, Blasco, Partridge, Serrano, & Kroemer, 2013); however, it remains elusive what are the crucial drivers for aging at cellular and molecular levels. An aged epidermis shows histological and functional changes, including a decreased proliferative capacity (Charruyer et al., 2009; Gilchrest, 1983) and lower success in epidermal engraftment (Piccin & Morshead, 2010), a decrease in epidermal thickness, flattening of epidermal–dermal junction (Changarathil, Ramirez, Isoda, Sada, & Yanagisawa, 2019; Giangreco, Goldie, Failla, Saintigny, & Watt, 2010; Langton, Halai, Griffiths, Sherratt, & Watson, 2016; Makrantonaki & Zouboulis, 2007), delayed wound healing (Keyes et al., 2016), decreased barrier function (Gonzales & Fuchs, 2017), increased risk of cancer (Adams, Jasper, & Rudolph, 2015; López‐Otín et al., 2013), impaired HF stem cell lineages (Matsumura et al., 2016), and interaction with their niche (Ge et al., 2020). Mutant mouse studies and transcriptome analyses have suggested that the age‐related epidermal dysfunction could be due to defects in IFE and HF stem cells to interact with other cell types or extracellular matrix in skin (Ge et al., 2020; Giangreco, Qin, Pintar, & Watt, 2008; Keyes et al., 2016; Liu et al., 2019; Matsumura et al., 2016; Watanabe et al., 2017). However, changes in gene expression at the transcription level may not fully explain the molecular aspects of stem cell aging in skin.

Glycosylation is a reaction that proteins or lipids are modified with glycans (Varki, 2009). The protein glycosylation involves stepwise addition and removal of glycans, primarily mediated by glycosyltransferases and glycosidases (Spiro, 2002). The presence of glycans determines the structure, stability, and localization of glycoproteins, which affect a wide variety of biological processes, such as development (Haltiwanger & Lowe, 2004), tumorigenesis (Ohtsubo & Marth, 2006) and inflammation (Varki & Gagneux, 2015). Glycans are required for stem cell regulations by modulating signaling molecules that govern self‐renewal and differentiation of stem cells (Nishihara, 2018). As glycans are located at the cell surface, they have been utilized as biomarkers, for example, pluripotent status of mouse embryonic stem cells (Adewumi et al., 2007; Muramatsu & Muramatsu, 2004; Muramatsu & Muramatsu, 2004) and human induced pluripotent stem cells (Tateno et al., 2011). Given the role of glycans in diverse biological and biochemical processes, glycosylation might play an important role in the process of stem cell aging. However, the glycosylation state of stem cells in aged mammalian tissues remains largely uncharacterized.

The glycome analysis of tissue stem cells has been challenging, as tissue stem cells are rare and large amounts of samples are required for the structural analysis of glycans by mass spectrometry. Lectin microarray, a platform for high‐throughput glycome analysis, enables a comprehensive glycan profiling even from a relatively small number of cells (Kuno et al., 2005). Lectins are a class of glycan‐binding proteins that recognize various glycan structures (Hirabayashi, 2004). In lectin microarrays, a series of lectins with various glycan‐binding specificities are immobilized on a glass slide (Hirabayashi, Yamada, Kuno, & Tateno, 2013). Lectin–glycan interactions are quantitatively measured as fluorescent signals after incubation with fluorescence‐labeled samples in the lectin microarray (Kuno et al., 2005). Using this technology, glycoproteins isolated from various biological samples can be utilized for glycome analysis without the liberation of glycans (Tateno et al., 2007).

In our current study, we performed a comprehensive glycome analysis of IFE and HF stem cells in the old mouse skin by using lectin microarray consisting of 96 lectins with various glycan‐binding specificities (Tateno et al., 2011). We found that epidermal stem cells undergo global changes in their glycosylation patterns during aging, with decreased mannose and increased sialic acid (Sia) modifications. By overexpressing glycogenes in vitro, we recapitulated the old‐type glycome patterns in epidermal stem cells, which led to a decline in the proliferation capacity. We thus propose functional implications of glycans in stem cell regulation.

## 2 RESULTS

### 2.1 Distinct glycosylation patterns between young and old epidermal stem cells

**FIGURE 1:**
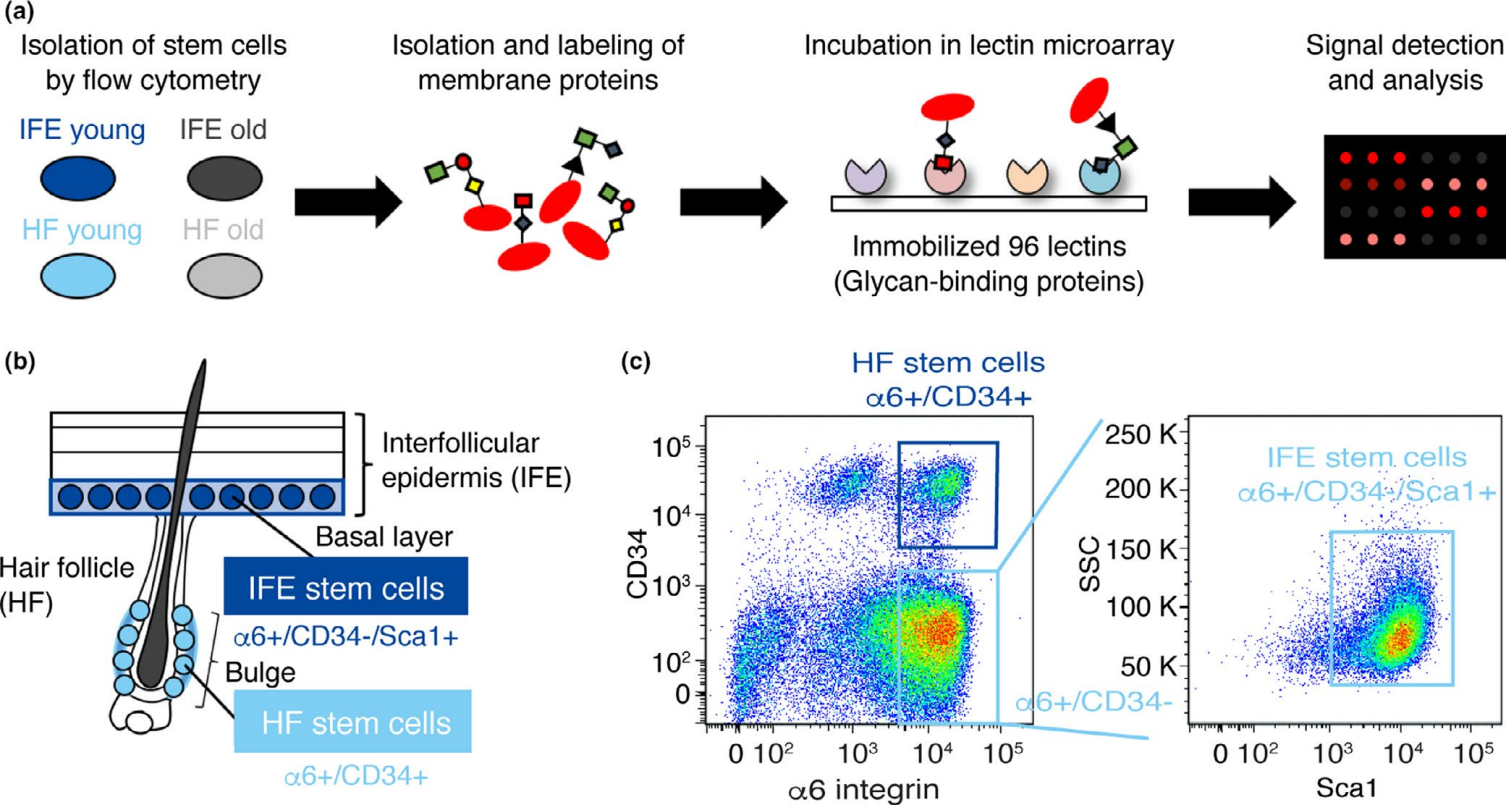
Schematic representation of lectin microarray using freshly isolated epidermal stem cells. (a) Schematic representation of lectin microarray analysis. Hydrophobic fractions containing membrane proteins are isolated, fluorescently labeled, and incubated with lectin microarray, in which 96 lectins are immobilized on glass slides. The lectin–glycan interactions are measured and quantified as signal intensities obtained from each lectin spot. (b) A schematic representation of epidermal cell types in mouse skin and cell surface markers used. (c) Flow cytometry dot plot and sorting gates for the isolation of skin epidermal subpopulations. Interfollicular epidermal (IFE) stem cells are defined as ⍺6‐integrin+/CD34−/Sca1+, and hair follicles (HF) stem cells are defined as ⍺6‐integrin+/CD34+.

**FIGURE 2:**
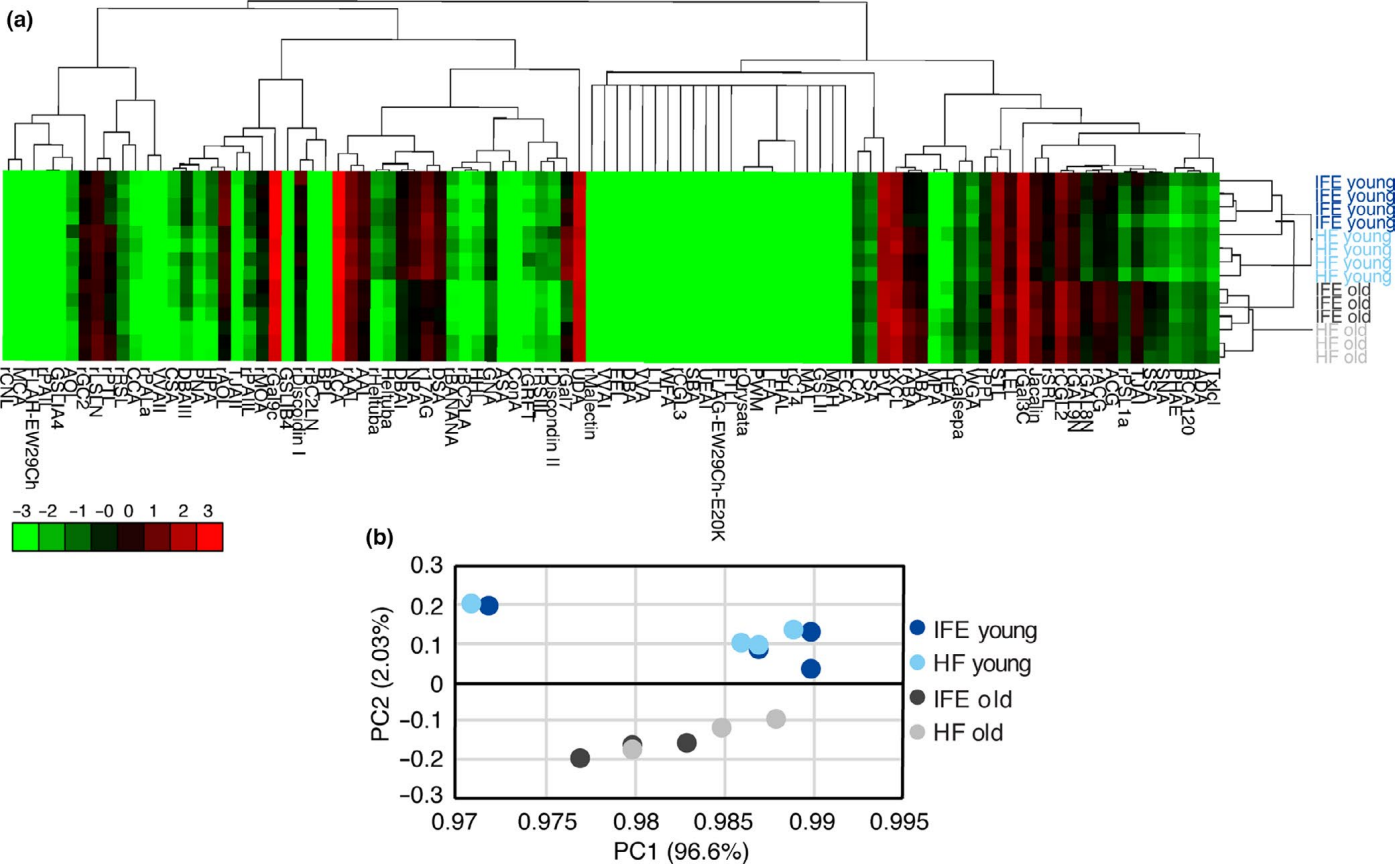
Glycome analysis of young and old epidermal stem cells. (a) Heat map and hierarchical clustering of lectin microarray signals. Each row represents different stem cell populations isolated from an individual mouse (*N* = 4 for young mice, *N* = 3 for old mice). Ninety‐six lectins are shown on columns. (b) Principal component analysis of the mean normalized signals obtained from lectin microarray. A scatter plot for principal component (PC) 1 and 2 is shown. Each dot represents the sample derived from an individual mouse. Different cell types are indicated by color.

To analyze the glycosylation state of epidermal stem cells during aging, IFE and HF stem cells were isolated from wild‐type C57BL/6 mice at 2 months (young, *N = 4)* and 22‐24 months (old, *N* = 3) of age and subjected to lectin microarray (Figure 1a). IFE stem cells (α6‐integrin+/CD34−/Sca1+) and HF stem cells (α6‐integrin+/CD34+) were separated by flow cytometry based on their differential expression of cell surface markers (Figure 1b,c). In old mouse skin, we detected significantly lower number of HF stem cells as previously reported (Matsumura et al., 2016), whereas the number of IFE stem cells remained unchanged (Figure S1). We asked whether their cell surface glycans were affected by aging. The hierarchical clustering of lectin microarray data showed that young and old samples were clustered separately, indicating their distinct glycosylation patterns (Figure 2a). Stem cells in different epidermal compartments, the IFE and HF, also showed differential glycosylation patterns, compatible with their distinct transcriptome signatures (Joost et al., 2016). To further examine the correlation of each cell population, we performed a principal component analysis (PCA) to dissect the similarity or differentiates among the samples. Young and old epidermal stem cells were separated by the biplot of principal components 1 and 2 (Figure 2b), supporting their differential lectin patterns. Thus, these data suggest that mouse IFE and HF stem cells undergo global alterations in glycosylation during aging.

### 2.2 Classes of lectins that differentially identified glycans in young and old stem cells

**FIGURE 3:**
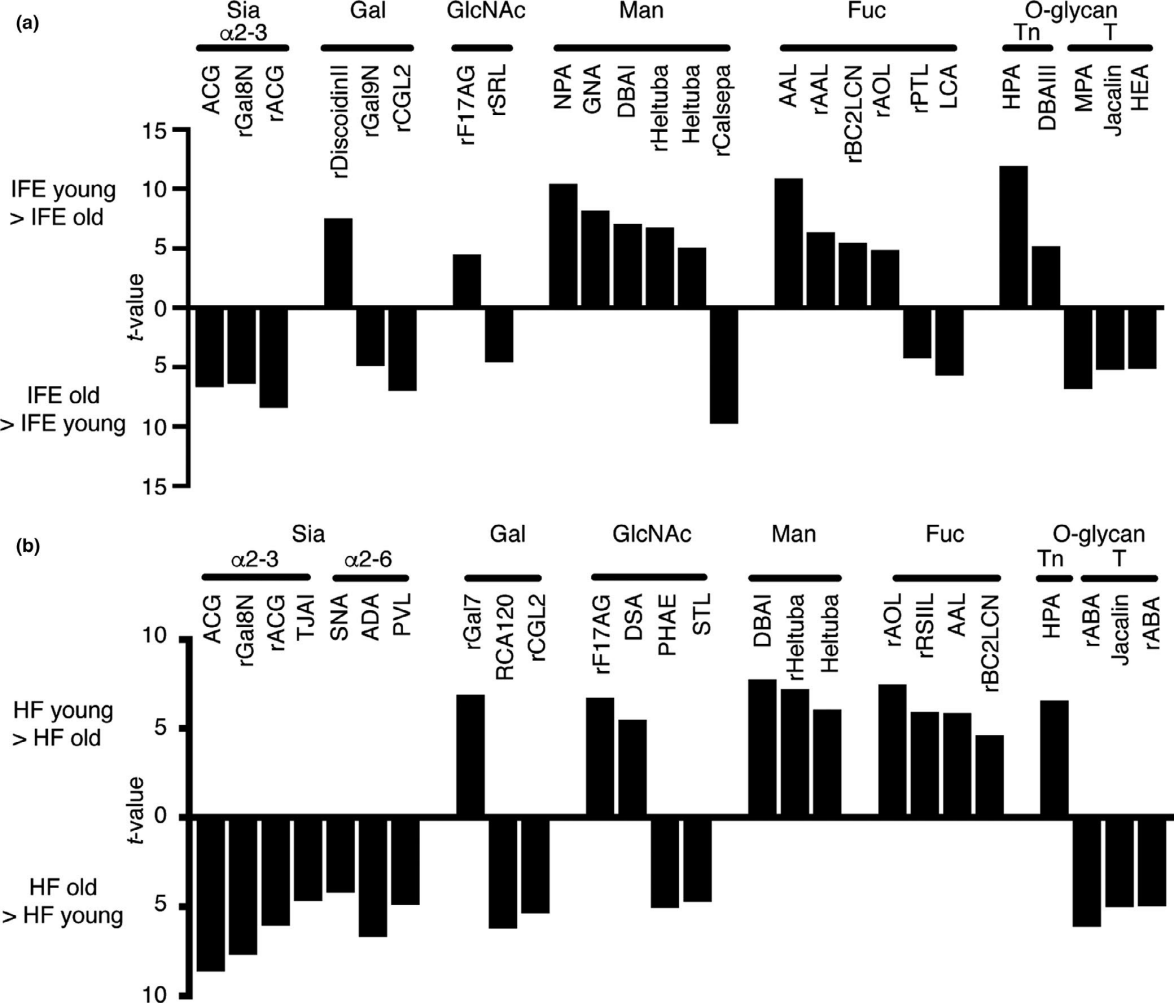
List of lectins significantly changed between young and old epidermal stem cells. (a, b) Lectins bound differentially to the young or old interfollicular epidermis (IFE) (a) and hair follicles (HF) (b). Statistically significant differences are calculated by unpaired Student's *t* test and *p* < 0.01 are selected. Lectins are categorized based on their binding specificities. Data are shown with *t*‐values. Also, see Table S1.

For the identification of lectins that were differentially bound to glycan structures between young and old stem cells, statistical analysis was performed using the mean normalized signals obtained from lectin microarray. Several classes of lectins were significantly changed (*p* < 0.01) between young and old stem cells (Figure 3 and Table S1), and we categorized them based on their glycan‐binding specificities.

In the IFE, 13 lectins were significantly higher in young stem cells, whereas 12 lectins were higher in old stem cells (Figure 3a and Table S1). The lectins enriched in young IFE stem cells included mannose‐binding lectins (NPA, GNA, DBAI, rHeltuba, Heltuba) (Maupin, Liden, & Haab, 2012; Tateno et al., 2011), fucose‐binding lectins (AAL, rAAL, rBC2LCN, rAOL) and O‐glycan (Tn)‐binding lectins (HPA, DBAIII). Consistently, it has been shown that high mannose‐type N‐glycans were highly enriched in human embryonic stem cells (An et al., 2012), with a possible role in the maintenance of stemness. The fucose (α1‐2)‐binding rBC2LCN has previously been identified as a lectin biomarker for undifferentiated pluripotent stem cells (Tateno et al., 2011).

In contrast, the lectins enriched in old IFE stem cells included Sia‐binding lectins (ACG, rACG, rGal8N) (Itakura et al., 2016; Sasaki, Itakura, & Toyoda, 2017), fucose (α1‐6)‐binding lectins (rPTL and LCA) and O‐glycan (T antigen) binding lectins (MPA, Jacalin and HEA) (Figure 3a). Since sialylation has been implicated in the aging of muscle and fibroblasts (Hanisch et al., 2013; Itakura et al., 2016; Sasaki et al., 2017), it might have an universal role in the process of aging. In HFs, 11 and 15 lectins showed significant enrichment in young and old stem cells, respectively (Figure 3b and Table S1). Notably, lectins of similar functional classes were detected with significant differences in both IFE and HFs. Taken together, our lectin microarray analysis identified common sets of lectins that recognize age‐dependent glycan changes in IFE and HF stem cells: decreased mannose‐binding lectins and increased Sia‐binding lectins during aging.

### 2.3 Old epidermal stem cells display decreased mannose and increased Sia modifications

**FIGURE 4:**
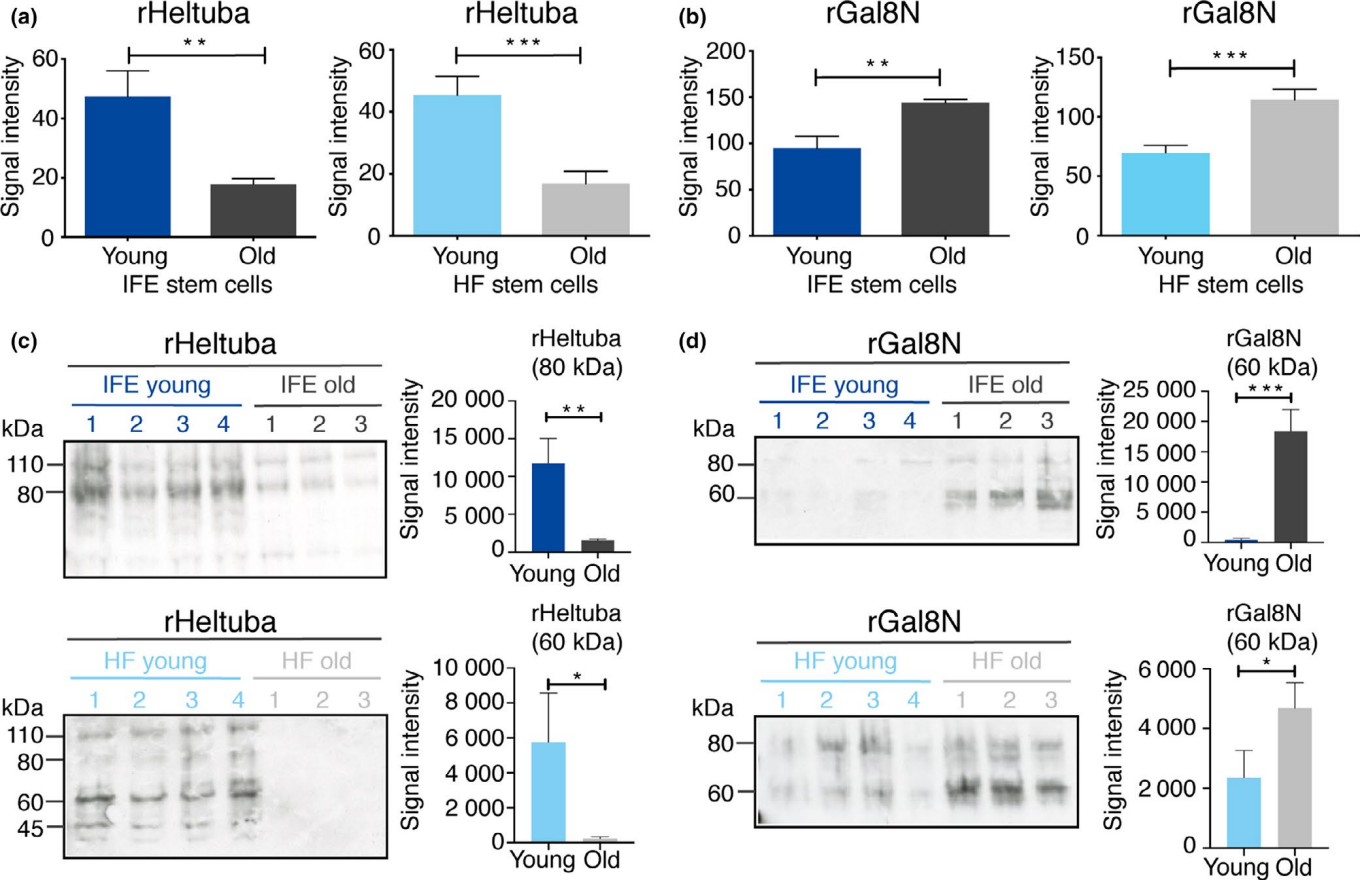
Detection of young and old epidermal stem cells by rHeltuba and rGal8N lectins. (a, b) Signal intensities of rHeltuba (Manα1‐3Man, Manα1‐6Man) (a) and rGal8N (α2‐3Sia) (b) in the lectin microarray are shown. The lectin signals of individual mice are averaged and normalized to the average of 96 lectins. *N* = 4 for young mice, *N* = 3 for old mice. Data are shown as means ±*SD*. Student's *t* test. ***p < 0.001. **p < 0.01. *p < 0.05. (c, d) Lectin blotting using the horseradish peroxidase (HRP)‐labeled lectins, rHeltuba (c) and rGal8N (d). Young and old stem cells in the interfollicular epidermis (IFE) and hair follicles are used. *N* = 4 for young mice, *N* = 3 for old mice. One microgram of protein from a single mouse is applied in each lane. The signal intensities of bands with indicated size are quantified.

To detect age‐related glycan changes in epidermal stem cells, a mannose‐binding rHeltuba and an α2‐3Sia‐binding rGal8N were selected as recombinant lectin probes for further analysis. Quantification of signals in lectin microarray confirmed significantly higher signals of rHeltuba in young stem cells compared with old stem cells both in the IFE and HFs (Figure 4a). In contrast, rGal8N showed higher signals in old stem cells than young stem cells (Figure 4b). These results were validated by lectin blotting. One microgram of membrane proteins was separated by SDS‐PAGE and blotted with two lectins conjugated with horseradish peroxidase (HRP). The lectin blotting using rHeltuba showed decreased signals in old IFE and HF stem cells compared with young counterparts (Figure 4c), consistent with lectin microarray results. The major bands were detected at 80 and 110 kDa in young IFE, and at 45 and 60 kDa in young HF in addition to 80 and 110 kDa bands, suggesting the mannose modification in multiple proteins (Figure 4c). In contrast, rGal8N showed higher signals in old stem cells compared with young stem cells and major bands around 60 and 80 kDa were detected (Figure 4d). Hence, the identified lectins, rHeltuba and rGal8N, successfully detected distinct glycosylation between young and old epidermal stem cells.

### 2.4 Detection of age‐related glycan changes in epidermal stem cells by flow cytometry using rHeltuba and rGal8N

**FIGURE 5:**
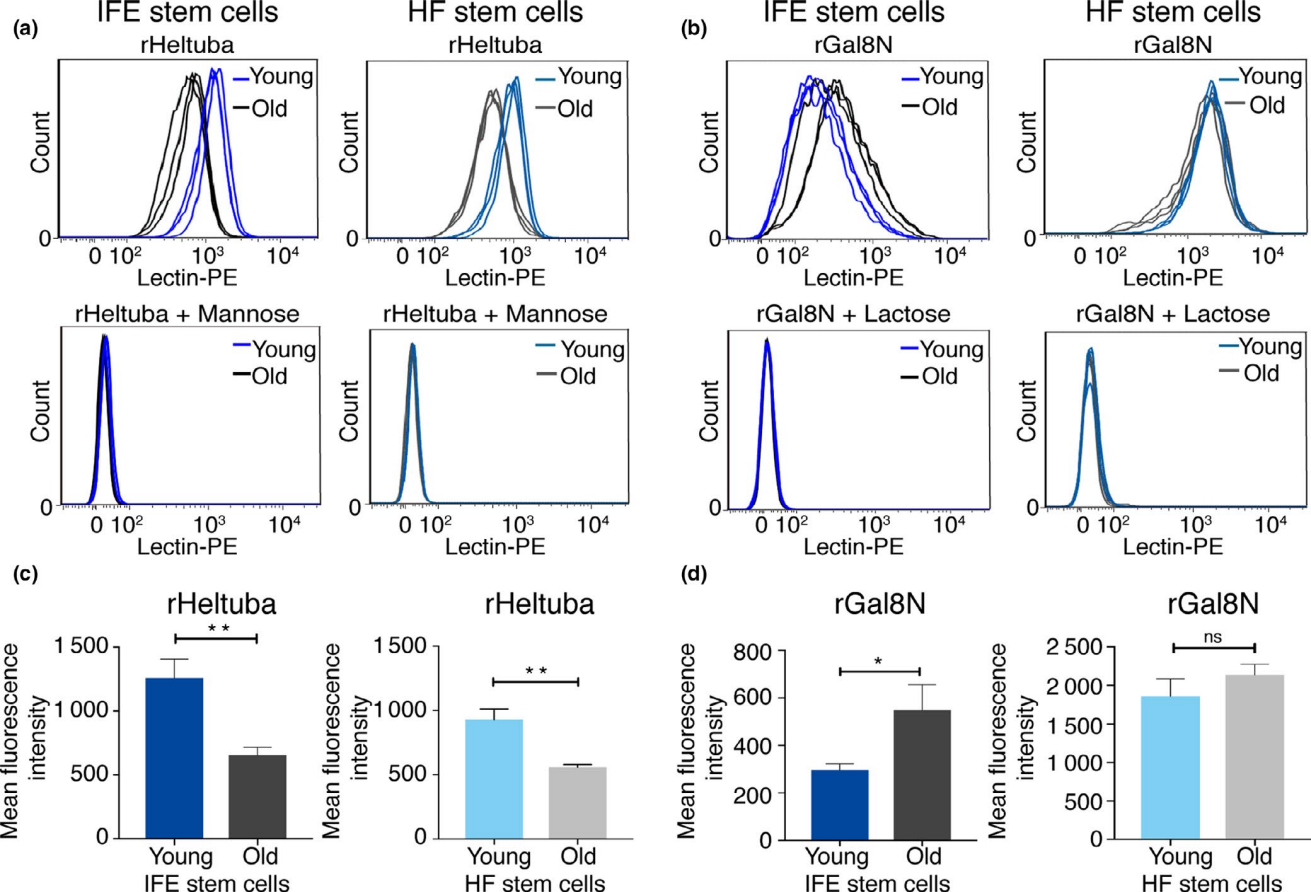
The rHeltuba and rGal8N lectins differentially bind to freshly isolated young and old stem cells. (a, b) The histogram shows signal intensities of PE‐labeled rHeltuba (1 µg/ml) (a) or rGal8N (10 µg/ml) (b) in the interfollicular epidermis (IFE) and hair follicles (HF) detected by flow cytometry. *N* = 3 for young and old mice. Data from each mouse are shown as an individual line. For inhibition of rHeltuba and rGal8N, 0.1 M mannose or 0.1 M lactose is used, respectively. (c, d) Quantification of mean fluorescence intensity obtained by flow cytometry. Data are shown as means ± *SD*. Statistical analysis is performed using the unpaired Student's *t* test. **p < 0.01. *p < 0.05. ns: not significant; rGal8N in HF stem cells, *p* = 0.1412.

To test the ability of lectin‐directed detection of glycans in living stem cells, we employed flow cytometry analysis in young and old stem cells using rHeltuba and rGal8N. Fluorescent‐labeled lectins (rHeltuba and rGal8N) were incubated with freshly isolated epidermal stem cells from wild‐type skin at 2 months (young, *N* = 3) or 22‐24 months (old, *N* = 3) of age. Flow cytometry analysis using rHeltuba showed a higher peak of signals in young stem cells compared with old stem cells in both IFE and HFs (Figure 5a, upper graphs). Statistical analysis of the mean fluorescence intensity of rHeltuba signals showed a significant difference between young and old HF stem cells (Figure 5c). To verify the specificity of rHeltuba binding to stem cells, competition assays were performed by adding excess mannose during incubation of the lectin with epidermal stem cells. Indeed, the rHeltuba signals in both young and old epidermal stem cells were abrogated in the presence of mannose (Figure 5a, lower graphs), confirming that the lectin detected mannose modifications on the surface of stem cells.

Similar flow cytometry experiments with rGal8N, the α2‐3Sia‐binding lectin, in the IFE showed a shift toward the higher signal intensity in old stem cells compared with young stem cells (Figure 5b, upper left, and 5d, left). The signals of rGal8N were inhibited by lactose, confirming the specific binding of rGal8N to glycans. In the HFs, however, there were no significant differences in the rGal8N signal intensity between young and old stem cells (Figure 5b, upper right, and 5d, right). One possible interpretation is that glycolipids, which content may differ between HF and IFE stem cells, had been detected by rGal8N in live HF cells and masked the difference between young and old HF stem cells. Taken together, these data indicate that both rHeltuba and rGal8N lectin probes successfully detected glycan changes in freshly isolated IFE stem cells by flow cytometry.

### 2.5 Upregulation of sialyltransferase and mannosidase genes in old epidermal stem cells

**FIGURE 6:**
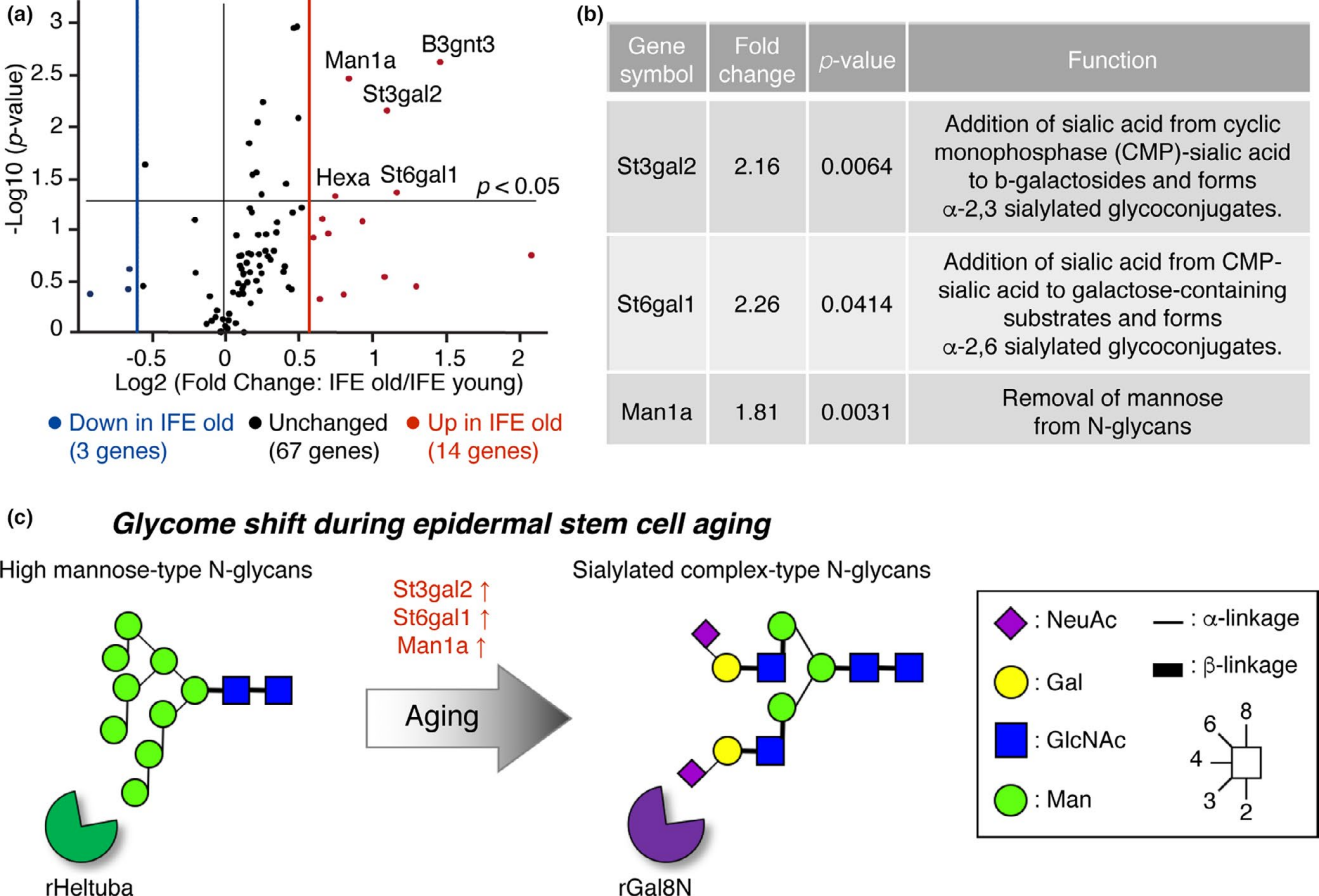
Gene expression analysis of glycosylation‐related genes using RT^2^ Profiler PCR array. (a) The volcano plot represents fold change and *p*‐values on *x*‐ and *y*‐axis, respectively. The vertical red and blue lines represent a fold‐change cutoff of ≥1.5. *N* = 3 for young mice, *N* = 3 for old mice. Also, see Table S2. (b) Lists of differentially expressed sialyltransferase and mannosidase genes. (c) Schematic representation of the putative glycan changes during epidermal stem cell aging.

To address which enzymes are responsible for age‐related glycosylation changes in epidermal stem cells, we performed gene expression analysis using RT^2^ profiler PCR array of mouse glycosylation‐related genes. RNAs isolated from IFE stem cells at 2 months (young, *N* = 3) or 22‐24 months (old, *N* = 3) of age were used for quantitative PCR. Among 84 genes involved in the glycosylation pathway, 14 genes were ≥1.5 fold upregulated in old IFE stem cells, whereas 3 genes were ≥1.5 fold downregulated (Figure 6a and Table S2). Among them, five genes were identified with statistically significant differences (*p* < 0.05). Sialyltransferase genes (*St3gal2* and *St6gal1*) were upregulated in old IFE stem cells (Figure 6a,b), consistent with our lectin microarray (Figure 3). St3gal2 catalyzes the transfer of Sia from cyclic monophosphate‐Sia to β‐galactosides and forms α‐2,3 sialylated glycoconjugates (Varki, 2009). Similarly, St6gal1 catalyzes the addition of Sia to a galactose‐containing substrate and form α‐2,6 sialylated glycoconjugates (Varki, 2009). We also found that mannosidase gene *Man1a* was increased in old stem cells (Figure 6b). Man1a is an α‐1,2 mannosidase and is responsible for the removal of mannose residues to initiate the complex‐type N‐glycan formation (Varki, 2009), which matches with the decreased signals of mannose‐binding lectins in old IFE stem cells (Figure 3). Similarly, we also found an increased expression of *Man1a*, *St3gal2*, *St6gal1* in the old HF stem cells (Figure S2 and Table S2). Thus, glycan changes of epidermal stem cells during aging are possibly mediated by the changes in glycosyltransferase and glycosidase expressions with age.

### 2.6 Recapitulation of old‐type glycosylation pattern by overexpressing glycogenes causes functional impairment of epidermal stem cells in vitro

**FIGURE 7:**
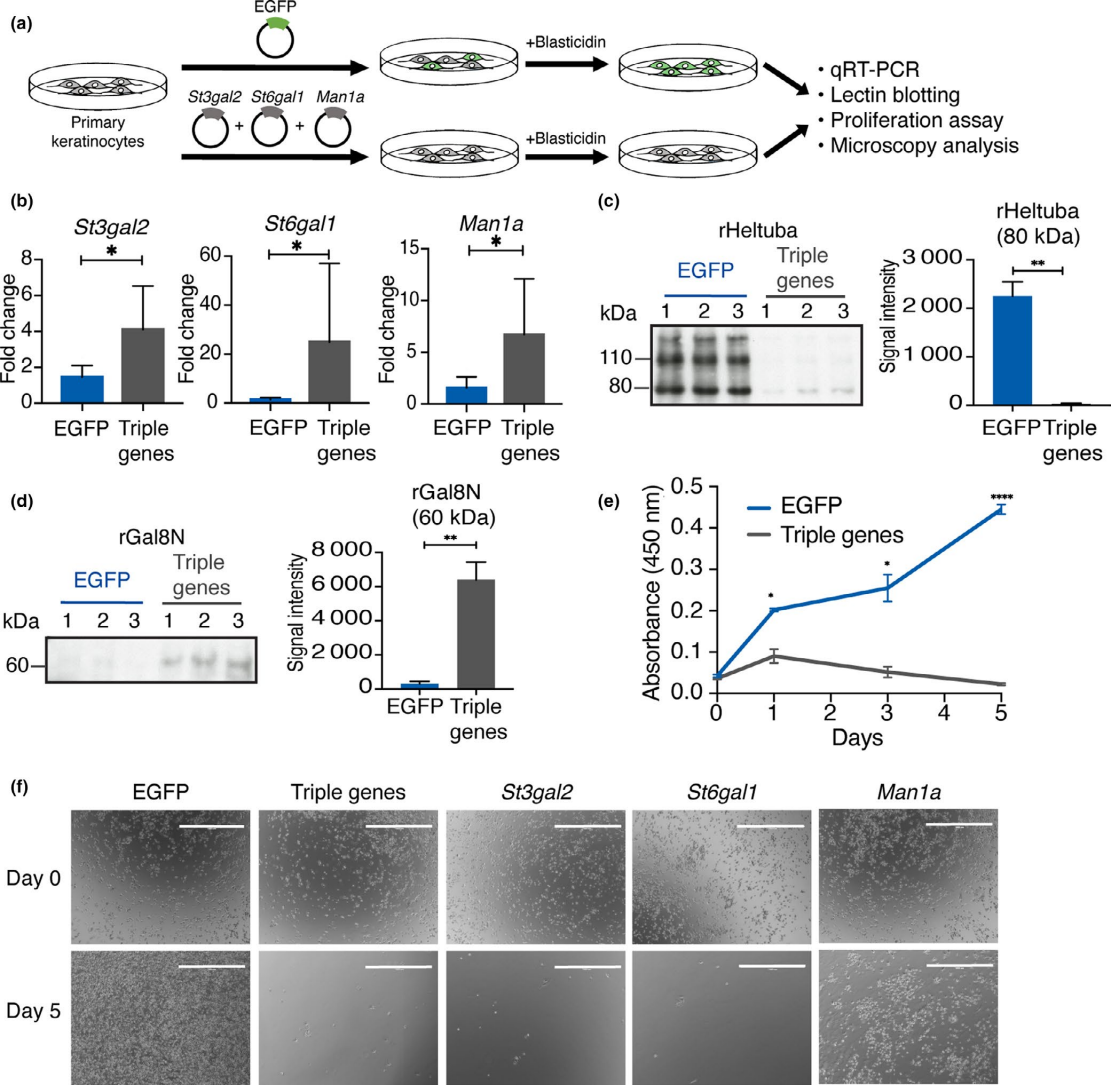
Aging‐associated glycogene overexpression leads to an impaired keratinocyte growth. (a) Scheme of the glycogene overexpression using the lentivirus system. (b) The qRT‐PCR of *Man1a*,* St3gal2*,* St6gal1* mRNA expression at 4 days after blasticidin selection (*N* = 3). Lenti‐EGFP is used as a control. Data are shown as means ± *SD*. Mann–Whitney test. *p < 0.05. (c, d) Confirmation of glycan changes by lectin blotting using the horseradish peroxidase (HRP)‐labeled lectins, rHeltuba (c) and rGal8N (d). One microgram of protein from three independent experiments is applied on each lane. Data are shown as means ± *SD*. Students *t* test. ***p < 0.001. **p < 0.01. *p < 0.05. The signal intensities of bands with indicated size are quantified. (e) Proliferation assay of primary keratinocytes after overexpressing glycogenes. The *x*‐axis represents the time points, and the *y*‐axis represents the absorbance at 450 nm. Absorbance is measured at 0, 1, 3, and 5 days post‐infection. Data are shown as means ± *SD*. Students *t* test. ***p < 0.001. **p < 0.01. *p < 0.05. (f) Representative images of the primary keratinocytes infected with lenti‐EGFP, or a combination or single lenti‐*Man1a*, ‐*St3gal2*, and ‐*St6gal1* at day 0 and 5.

Finally, we addressed whether age‐related glycan changes are a consequence of aging or causal to induce age‐related phenotypes in epidermal stem cells. To mimic the glycosylation pattern of old epidermal stem cells, we overexpressed three glycogenes (*Man1a*,* St3gal2*,* St6gal1*) in primary epidermal keratinocytes, an in vitro model of epidermal stem cells, and modified cell surface glycans to aging‐like status (Figure 7a). Successful gene overexpression and changes of glycosylation were evaluated by qRT‐PCR (Figure 7b) and lectin blotting (Figure 7c,d). The keratinocytes showed decreased mannose and increased Sia modifications (Figure 7c,d), which are similar to the glycosylation pattern of old epidermal stem cells in vivo (Figure 4). Overexpression of three glycogenes resulted in significantly less ability to proliferate as compared to the control keratinocytes infected with EGFP, and detached from a dish within 5 days of culture (Figure 7e,f). The overexpression of *Man1a* alone showed milder effects than *St3gal2* or *St6gal1* alone (Figure 7f). These data indicate that age‐related glycan changes may in part be responsible for a decline in the proliferation ability of epidermal stem cells during aging.

## 3 DISCUSSION

In vivo sign of aging in the skin can be observed at the tissue and organismal levels; however, the molecular aspects of aging at the stem cell level remains elusive. In our current study, we performed a high‐throughput lectin‐based glycan profiling on murine epidermal stem cells and revealed their dynamic glycan alterations during aging. We propose a concept, “glycome shift” as a new molecular factor of epidermal stem cell aging (Figure 6c): high mannose‐type N‐glycans are globally replaced by α2‐3/6 sialylated complex‐type N‐glycans with age. Intriguingly, overexpression of three glycogene(s) (*Man1a*,* St3gal2*,* St6gal1*) recapitulated the aging glycan patterns and impaired the growth of primary keratinocytes, suggesting that the glycans could be one of the drivers of age‐related decline in the proliferation ability of epidermal stem cells. The identified lectins, the mannose‐binding rHeltuba and the α2‐3Sia‐binding rGal8N can be used as probes to visualize, select, or remove aged stem cells, with implications in future applications for regenerative therapy and diagnosis of skin aging. We also provide a proof of concept that our lectin microarray platform (Tateno et al., 2011) can successfully analyze the glycome of adult tissue stem cells, which are rare in tissue (≤1% of total skin cells) and their biochemical properties are not well‐characterized due to technical difficulties.

As glycosylation plays a critical role in cell–cell and cell–matrix interactions, the changes in glycans on the surface of epidermal stem cells might affect their ability to interact with neighboring stem cells, other cell types (e.g., fibroblasts, immune cells, and blood vessels), basement membrane and signaling molecules, all of which are essential components for maintaining the skin integrity. It will be interesting in the future to identify core proteins in which differential glycosylation takes place and to reveal the functional importance and biological meaning of glycosylation in age‐related skin dysfunction.

An aged skin exhibits declined wound healing ability, which is in part caused by impaired crosstalk between epidermal stem cells and dendritic epithelial T cells (Keyes et al., 2016). Given that several immune cells, including dendritic cells, have mannose‐binding receptors in the epidermis (Wollenberg et al., 2002), the decreased mannose in old IFE stem cells that we observed here (Figure 6c) could be associated with the defective stem cell–immune cell interaction in aged skin.

Our study showed an increase in α2‐3 and α2‐6 sialylation along with the expression of the corresponding sialyltransferase (*St3gal2* and *St6gal1*) in old IFE stem cells (Figure 6c). In agreement with our findings, sialylation was reported to be increased in the aged mouse muscle (Hanisch et al., 2013). The upregulation of sialyltransferases has also been suggested as a potential aging marker in human, which shows a higher activity of *St6gal1* in the plasma of individuals above 80 years of age (Catera et al., 2016). In addition, an α2‐6 sialylation and the expression of *St6gal1* were upregulated during epithelial to mesenchymal transition and tumor formation (Lu et al., 2014; Swindall et al., 2013). By contrast, α2‐3/6 sialylation was reported to be decreased during senescence and aging of human dermal fibroblasts (Itakura et al., 2016). In human pluripotent or mesenchymal stem cells, a higher sialylation is associated with a greater potential of stem cells (Hasehira et al., 2012; Tateno et al., 2011; Wang et al., 2015). The observed differences in the sialylation patterns might be due to the differences in cell types, species, or target proteins, indicating a diverse role of sialylation in the process of aging. Future studies using conditional knock‐out or overexpression of differentially expressed glycosyltransferases in the mouse epidermis will directly address the role of sialylation in the context of epidermal stem cell aging.

## 4 EXPERIMENTAL PROCEDURES

### 4.1 Mice

All animal procedures were conducted following animal experimentation guidelines approved by the Institutional Animal Experiment Committee at the University of Tsukuba. Young (2‐month‐old) and old (22‐24‐month‐old) C57BL/6 mice were purchased from Charles River Laboratories or Japan SLC. Both male and female mice were used for experiments. All the experimental mice were housed in Laboratory Animal Resource Center, University of Tsukuba prior to experiments.

### 4.2 Isolation of epidermal stem cells by flow cytometry

Mouse dorsal and ventral skin were dissected and the subcutaneous and fat tissues were removed from the dermal side of the skin. The skin was incubated in 0.25% trypsin/versene overnight at 4°C and for 30 min at 37°C. The single‐cell solution was prepared by scraping the epidermis and subsequent filtering with strainers (70 μm, followed by 40 μm). Cells were stained with the following antibodies for 30 min on ice: CD34‐biotin (1:50, eBioscience), Streptavidin‐APC (1:100, BD Biosciences), α6‐integrin‐BUV395 (1:100, BD Biosciences, custom order) and Sca1‐BV421 (1:100, BD Biosciences). The dead cells were excluded by staining with propidium iodide (Sigma‐Aldrich). Cell isolation was performed with FACS Aria (BD Biosciences), and the data were analyzed with the FlowJo software (BD, Franklin Lakes, NJ).

### 4.3 Membrane protein isolation and quantification

Hydrophobic fractions containing membrane proteins were prepared using the CelLytic MEM Protein Extraction kit (Sigma‐Aldrich) following the manufacturer's protocols. Proteins were quantified using a micro BCA assay kit (Thermo Fisher Scientific). Protein amounts ranging from 15 to 30 μg were obtained from 100,000–300,000 IFE or HF stem cells.

### 4.4 Lectin microarray

The high‐density lectin microarray was produced according to the method previously described (Tateno et al., 2011). The protein concentration was adjusted to 2 μg/ml with PBST [10 mM PBS (pH 7.4), 140 mM NaCl, 2.7 mM KCl, 1% Triton X‐100] and was labeled with Cy3‐N‐hydroxysuccinimide ester (GE Healthcare). Cy3‐labeled proteins were diluted with probing buffer [25 mM Tris‐HCl (pH 7.5), 140 mM NaCl, 2.7 mM KCl, 1 mM CaCl_2_, 1 mM MnCl_2_, and 1% Triton X‐100] to 0.5 μg/ml and were incubated with the lectin microarray at 20°C overnight. Samples were washed with probing buffer for three times, and fluorescence images were captured using a Bio‐Rex scan 200 evanescent‐field‐activated fluorescence scanner (Rexxam Co. Ltd.).

The obtained signals were mean‐normalized and subjected to unsupervised hierarchical clustering, followed by a heat map analysis. The lectin signals of triplicate spots were averaged for each sample and normalized relative to the mean value of 96 lectins. The mean normalized lectin microarray data were used for unsupervised clustering with the average linkage method using Cluster 3.0 software. The heat map with clustering was visualized using Java TreeView. Significant differences in lectin intensity were calculated by unpaired Student's *t* test. The principal component analysis was performed by using the mean normalized signals and was generated using IBM SPSS Statistics software (IBM Japan, Ltd.). Principal component analysis (PCA) was run on the mean normalized intensity of the 96 lectins of the lectin microarray obtained from the different populations of epidermal stem cells from young (*N* = 4) and old (*N* = 3) mouse samples. After which, a biplot graph was plotted using the first two components.

### 4.5 Lectin blotting

Recombinant lectins (rHeltuba, rGal8N) were prepared using *Escherichia coli* as previously described (Tateno et al., 2011). Lectins were conjugated with HRP by using HRP labeling kit (Dojindo, Rockville, MD) at the concentration of 0.5 mg/ml and adjusted to the final concentration for incubation at 0.1 μg/ml.

One microgram of proteins from each cell population was separated by SDS‐PAGE on a 5‐20% gel (Perfect NT Gel system, NTH‐676HP, DRC, Tokyo, Japan) and transferred onto polyvinylidene fluoride membranes (Millipore, Burlington, MA). After blocking the membrane in Carbo‐Free blocking solution (Vector Laboratories, Burlingame, CA) for 1 hr at room temperature, it was incubated with HRP‐conjugated lectins overnight at 4°C. The signals were detected by using Western Lighting Plus (NEL104001EA, PerkinElmer). Lectin blot intensities were quantified using ImageJ software (National Institute of Health). The high‐intensity band was selected for quantification. Statistical significance was calculated by unpaired Student's *t* test (GraphPad Prism8 software).

### 4.6 Detection of lectin binding to epidermal stem cells by flow cytometry

Recombinant lectins (rHeltuba, rGal8N) were labeled with R‐Phycoerythrin (PE) using Phycoerythrin Labeling Kit ‐ NH2 (Dojindo) according to the manufacturer's protocol. The single‐cell solution was prepared as described above and resuspended in 1% BSA (Sigma‐Aldrich, A3059) without using the serum. Cells were stained with Lectin‐PE for 1 hr at 4°C, at the following concentrations: 1 μg/ml for rHeltuba‐PE and 10 μg/ml for rGal8N‐PE. For the inhibition assays, 0.1 M D‐(+)‐Mannose (Sigma‐Aldrich, M2069) and 0.1 M lactose monohydrate sugar (Wako Pure Chemical Industries, 121‐00105, Ltd) were used. After washing, cells were stained with antibodies for 30 min on ice and analyzed by FACS Aria (BD Biosciences). Student's *t* tests were performed to compare lectin signal intensity of young versus old stem cells by using GraphPad Prism8 software.

### 4.7 RT^2^ profiler mouse glycosylation PCR arrays

Total RNAs were isolated using the RNeasy micro kit (QIAGEN), according to the manufacturer's protocol. The integrity of the isolated RNA was assessed by using RNA Pico Chips and Agilent 2100 bioanalyzer (Agilent Technologies). RNA samples with the RNA integrity number above 8 were used for further analysis. The cDNA from IFE and HF stem cells were synthesized from 50 and 5 ng of mRNA, respectively, using the RT^2^ PreAMP cDNA Synthesis Kit (QIAGEN, 330451) followed by pre‐amplification using the pathway‐specific primer mix for mouse glycosylation (QIAGEN, PBM‐046Z).

The relative mRNA expressions of 84 genes regulating mouse glycosylation were analyzed using RT^2^ Profiler™ PCR Arrays (QIAGEN, PAMM‐046Z) according to the manufacturer's instructions. The cDNA template prepared above was mixed with RT^2^ SYBR Green qPCR Master Mix (QIAGEN, 330501) and nuclease‐free water. The cDNA mixture of 25 μl was applied to each well of the PCR arrays that contain the preloaded primer mix for each gene. The real‐time PCR amplification and detection were performed using a Bio‐Rad CFX96 Touch™ Real‐Time PCR Detection System (Bio‐Rad). Amplification cycle was used as following: activation of DNA Taq polymerase at 95°C for 10 min, followed by 40 cycles of denaturation at 95°C for 15 s and annealing for 1 min at 60°C. The threshold cycle (C_t_) was used for PCR array quantification. The threshold values were set similarly across all the PCR array used in the analysis, and the baseline was defined by using the automated baseline option of the machine. Gene whose C_t_ cycle was more than 35 was set as undetectable. C_t_ values of biological replicates obtained from the real‐time PCR array analysis were used for the ∆∆ C_t_ –based fold‐change calculations. For the data analysis, web‐based PCR data analysis provided from the data analysis center of QIAGEN was used. Samples were normalized using automatic normalization from the five housekeeping genes (*Actb*,* B2m*,* Gapdh*,* Gusb*,* Hsp90ab1*) in the PCR array. An appropriate correction was also made during the web‐based data analysis for the pre‐amplification step. Gene expression whose fold is greater than 1.5 was selected.

### 4.8 Mouse primary keratinocytes isolation and culture

Mouse primary keratinocytes were isolated from 2‐day‐old C57BL6/J mouse skin as previously reported (Lichti, Anders, & Yuspa, 2008). Keratinocytes were seeded on mitomycin‐treated, mouse embryonic fibroblasts and grown in low‐calcium E‐medium (15% chelex‐treated FBS, 0.05 mM CaCl_2_). Keratinocytes were used from passage 6‐13 for the subsequent analysis.

### 4.9 Lentivirus production and infection

Mouse cDNA encoding *Man1a*, *St3gal2*, *St6gal1*, or EGFP were cloned into CSII‐CMV‐MCS‐IRES2‐Bsd vector (RIKEN, Tsukuba, Japan) and transfected to 293 T cells using Lipofectamine 3000 (Thermo Fisher Scientific) together with packaging vectors pRSV‐Rev, pMD2.G and pMDLg/pRRE (Addgene). The medium containing lentivirus was collected on day two and three post‐transfection and concentrated using a Lenti‐X concentrator (Takara Bio).

Mouse keratinocytes were seeded at 50,000 cells in a 24‐well culture plate coated with collagen IV (Sigma). One day later, keratinocytes were infected with lentivirus along with 4 µg/mL polybrene for 16 hr. The 300 µl of medium containing 100 µl each of the glycogenes (*Man1a*,* St3 Gal2*,* St6gal1*) or 300 µl of medium containing lenti‐EGFP were used. The medium was changed after 16 hr and the infected keratinocytes were selected by using blasticidin at a concentration of 1 µg/ml.

### 4.10 qRT‐PCR

Mouse keratinocytes RNA was isolated using the RNeasy micro kit (QIAGEN) and real‐time RT‐PCR was performed using iTaq Universal SYBR green supermix (Bio‐Rad) with the following primers; *Man1a* forward: 5′‐GAGACCCAGTCTTTGCCGAA‐3′, *Man1a* reverse: 5′‐CGACACATGATGTTGACCCC‐3′, *St3gal2* forward: 5′‐CCTAATGTGGATTGCCAGCG‐3′, *St3gal2* reverse: 5′‐TCTGGACCTTCTCTTTGTCCA‐3′, *St6gal1* forward: 5′‐GGGCACAAAAACTACCATCCG‐3′, *St6gal1 *reverse: 5′‐TGATACCACTGCGGAATGTCT‐3′.

### 4.11 Cell proliferation assay

Keratinocyte proliferation was measured by using the Cell‐Counting Kit‐8 (CCK‐8, Dojindo, Japan) following the manufacturer's instructions. In brief, blasticidin‐selected keratinocytes were seeded in triplicate in a collagen‐IV‐coated flat‐bottom 96‐well plate at 2,000 cells/well. Keratinocytes were grown in the E‐medium and analyzed at 0, 1, 3, and 5 days after infection. Ten microliter of CCK‐8 reagent was incubated for 2 hr, and the absorption of the samples was measured at 450 nm using xMark microplate reader (Bio‐Rad).

For microscope analysis, blasticidin‐selected keratinocytes were seeded at 5,000 cells/well in a collagen‐IV‐coated 24‐well plate. Images were acquired by using the Evos FL cell imaging system (Thermo Fisher Scientific) at indicated time points.

## CONFLICT OF INTEREST

The authors declare no conflict of interest.

## AUTHOR CONTRIBUTIONS

A.S. and H.Y. conceptualized the project. A.S. provided knowledge and techniques for stem cell analysis. H.T. provided knowledge and techniques of lectin analysis. A.S., H.T., L.O., and E.R. designed the experiments. L.O., G.C., A.S., E.R., and Y.X.N. performed experiments and analyzed the results. A.S., H.T., and H.Y. interpreted the results and supervised the project. L.O., A.S., H.T., and H.Y. wrote the manuscript. A.S. acquired funding.

## Supporting information

Fig S1

Fig S2

Table S1

Table S2
