# Supplementary figures and images for: Glycome profiling by lectin microarray reveals dynamic glycan alterations during epidermal stem cell aging
Source: Aging Cell. 2020 Jul 18;19(8):e13190. doi: 10.1111/acel.13190 (PMC7431822; doi:10.1111/acel.13190)

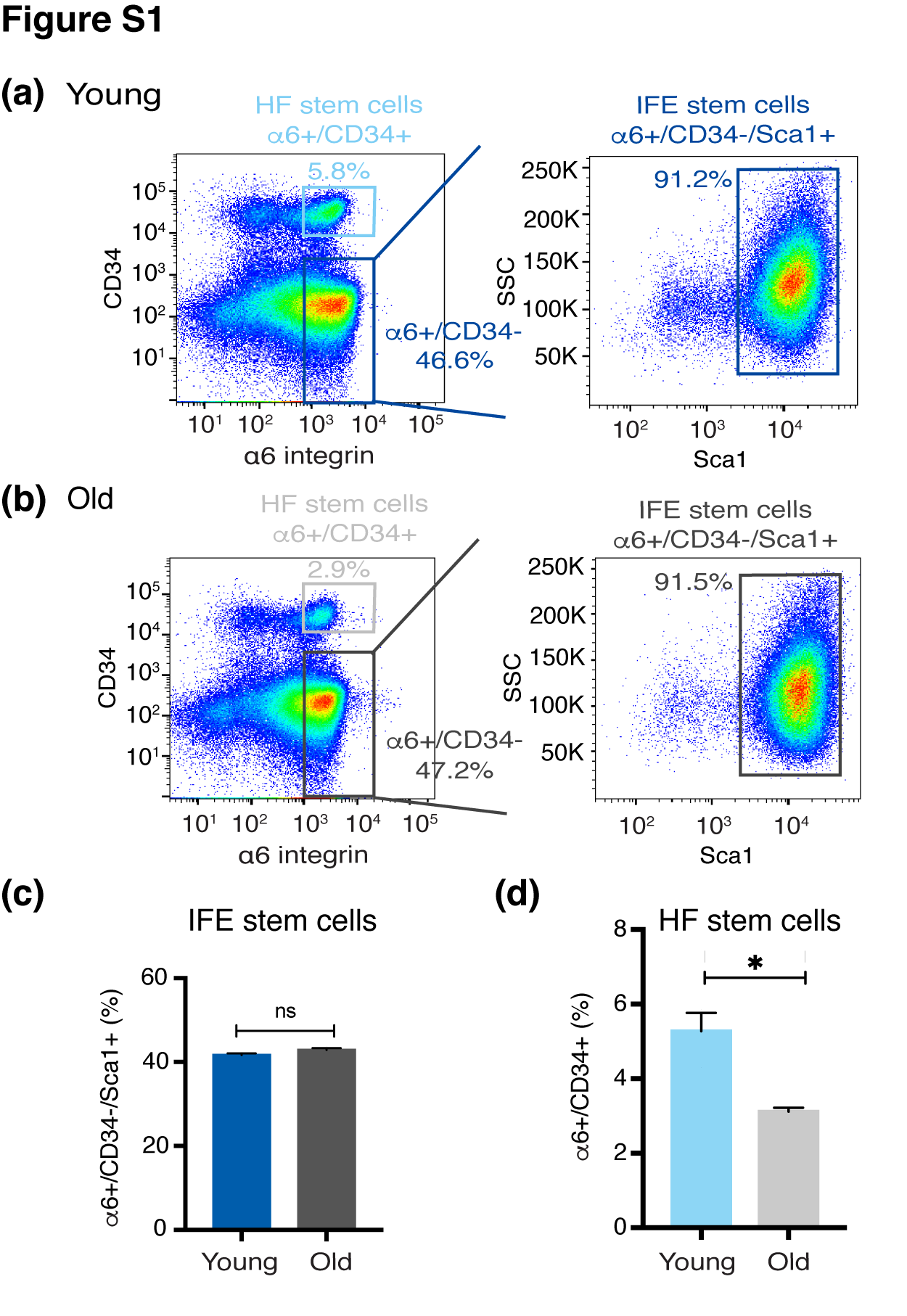

Supplement: Supplementary file 1 — Fig S1 [file ACEL-19-e13190-s001.tif]

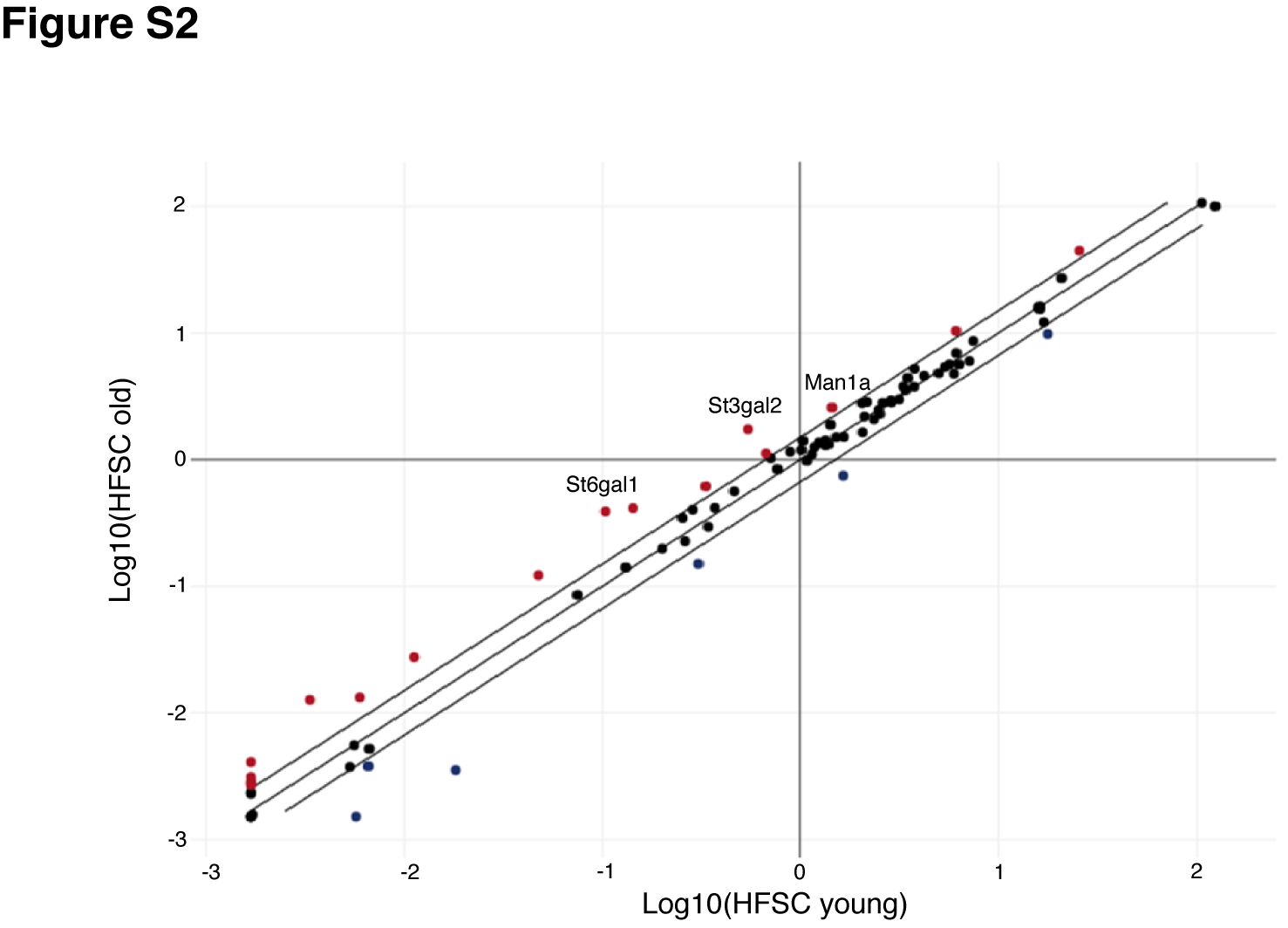

Supplement: Supplementary file 2 — Fig S2 [file ACEL-19-e13190-s002.tif]
